# Correction

**DOI:** 10.1093/bjd/ljae157

**Published:** 2024-05-06

The below-listed manuscripts were originally published without their graphical abstracts. This error has been corrected online for manuscripts ljad156 to ljad419. Any resulting pagination changes have been marked via lettered additional pages. For manuscripts ljae038 and ljae043, the error has been corrected in both the online and print versions.

- https://doi.org/10.1093/bjd/ljad156
- https://doi.org/10.1093/bjd/ljad220
- https://doi.org/10.1093/bjd/ljad252
- https://doi.org/10.1093/bjd/ljad281
- https://doi.org/10.1093/bjd/ljad305
- https://doi.org/10.1093/bjd/ljad369
- https://doi.org/10.1093/bjd/ljad399
- https://doi.org/10.1093/bjd/ljad402
- https://doi.org/10.1093/bjd/ljad419
- https://doi.org/10.1093/bjd/ljae038
- https://doi.org/10.1093/bjd/ljae043

